# Evaluation of the Antitumor and Antiproliferative Potential of Synthetic Peptides Derived from IsCT1, Associated with Cisplatin, in Squamous Cell Carcinoma of the Oral Cavity

**DOI:** 10.3390/molecules30122594

**Published:** 2025-06-15

**Authors:** Laertty Garcia de Sousa Cabral, Cyntia Silva de Oliveira, Vani Xavier Oliveira, Ellen Paim de Abreu Paulo, Jean-Luc Poyet, Durvanei Augusto Maria

**Affiliations:** 1 Laboratory of Development and Innovation, Butantan Institute, Sao Paulo 05585-000, Brazil; laertty.c@usp.br (L.G.d.S.C.); ellen.paulo.esib@esib.butantan.gov.br (E.P.d.A.P.); 2 Faculty of Medicine, University of Sao Paulo (FMUSP), Sao Paulo 05508-220, Brazil; 3 Department of Molecular Biology, Federal University of Sao Paulo (UNIFESP) Sao Paulo 09913-030, Brazil; cyntia.oliveira@unifesp.br (C.S.d.O.); vani.junior@ufabc.edu.br (V.X.O.J.); 4 Center for Natural and Human Sciences, Federal University of ABC, Santo Andre 09210-580, Brazil; 5 INSERM UMRS1342—CNRS EMR8000, Institut De Recherche Saint-Louis, Hôpital Saint-Louis, 75010 Paris, France; 6 Université Paris Cité, 75006 Paris, France

**Keywords:** tongue cancer, squamous cell carcinoma, synthetic peptide, anticancer peptide, apoptosis

## Abstract

Head and neck squamous cell carcinoma (SCC), particularly in the oral cavity, is among the most prevalent and lethal forms of cancer globally. Current therapeutic strategies, predominantly involving cisplatin, face challenges like chemoresistance and toxicity to normal cells, justifying the exploration of new approaches. This study evaluates the antitumor, antiproliferative, and immunomodulatory potential of a synthetic peptide derived from IsCT1 (Isalo scorpion cytotoxic peptide), named AC-AFPK-IsCT1, in combination with cisplatin in oral squamous cell carcinoma cellular models. Tumor and normal cells were treated with varying concentrations of cisplatin and peptide, and the cytotoxicity was measured through an MTT assay, while apoptosis and cell cycle alterations were assessed via flow cytometry. Interestingly, the combination of AC-AFPK-IsCT1 with cisplatin exhibited higher specificity for tumor cells, significantly reducing IC50 values compared to cisplatin used as a single agent. Moreover, the combination treatment induced pronounced S-phase cell cycle arrest and enhanced apoptotic activity, evidenced by the upregulation of caspase-3, caspase-8, and p53, while maintaining low toxicity in normal fibroblast cells. The peptide also modulated the mitochondrial membrane potential, further contributing to the activation of intrinsic apoptotic pathways. The data suggest that AC-AFPK-IsCT1 potentiates the antitumor effects of cisplatin by engaging both intrinsic and extrinsic apoptotic pathways while preserving normal cell viability. These findings underscore the potential of combining cisplatin with AC-AFPK-IsCT1 as a promising therapeutic strategy for improving the efficacy of chemotherapy in SCC, reducing systemic toxicity, and overcoming chemoresistance.

## 1. Introduction

The oral cavity represents the most common location for head and neck cancer, ranking as the eighth most prevalent form of cancer worldwide, with a higher frequency in developing nations [1]. Oral squamous cell carcinoma (SCC) stands as a primary cause of morbidity and mortality within head and neck cancer, constituting more than 90% of all oral cancer cases. The principal causative factors are alcohol and tobacco use, diet, viruses, radiation, genetic predisposition, or immunosuppression, among others [2].

Early diagnosis is frequently achievable, but advanced disease presentation remains common. The standard approach involves primary surgical resection, possibly followed by adjuvant therapy, with combined radiation and chemotherapy showing improved tumor-free survival statistics [3]. However, many head and neck cancers exhibit mutations in genes like p53, which result in cell cycle dysregulation, heightened invasiveness, and resistance to chemotherapy [4].

The 5-year survival rate of SCC slightly exceeds 50%, but radiation and chemotherapy may lead to functional deficits, such as osteoradionecrosis, xerostomia, mucositis, trismus, radiation caries, dysphagia, and altered periodontal ligaments [5]. The implant loss rate in irradiated bone is approximately 10% within three years. Consequently, there is a demand for therapies displaying increased specificity for tumor cells while reducing the systemic side effects linked to conventional treatments [6].

Cisplatin is one of the main chemotherapeutic agents used in the treatment of oral cavity cancer [7]. Despite a consistent rate of initial responses, cisplatin treatment often leads to the development of chemoresistance, resulting in therapeutic failure. There are at least two mechanisms by which tumor cells reduce the cytotoxic potential of cisplatin by inhibiting its binding to cytoplasmic targets and DNA [8]. The first mechanism involves a reduced intracellular accumulation of cisplatin, while the second mechanism entails increased sequestration of cisplatin via reduced glutathione, metallothionein, and other cytoplasmic scavengers with nucleophilic properties [9].

Peptides offer a promising substitute for traditional chemotherapy in cancer therapy [10]. They provide heightened target specificity and efficient intracellular transport, potentially mitigating the chemotherapy-associated drawbacks while augmenting the therapeutic specificity [11].

The IsCT peptide, extracted from the venom of the scorpion *Opisthacanthus madagascariensis*, is a short α-helical antimicrobial peptide. Scorpion venom represents an abundant source of biologically active compounds, particularly blockers of ion channels [12]. IsCT exhibits substantial antimicrobial capabilities and has displayed antitumor effects in various cancer cell lines, reducing migration in breast cancer and displaying cytotoxicity in chronic myeloid leukemia cells [13]. However, its severe hemolytic activity hampers IsCT clinical application.

Developing synthetic counterparts of natural peptides is a hopeful approach to mitigate general toxicity while retaining or even enhancing their therapeutic potential [14]. The current investigation aims to assess the antitumor, antiproliferative, and immunomodulatory attributes of a synthetic peptide derived from IsCT that we recently constructed, known as AC-AFPK-IscT1, in combination with cisplatin, using cellular models of tongue squamous cell carcinoma.

## 2. Results

### 2.1. Enhanced Cytotoxicity of Cisplatin Combined with AC-AFPK-IsCT Peptide in Tumor Cells

Viability assays indicate that tongue squamous cell carcinoma SCC-9 cells are sensitive to cisplatin, with IC50 values of 94.2 µM for a 24 h treatment and 5.2 µM for a 48 h treatment (Table 1; Supplemental Figure S2). Interestingly, the combination of cisplatin with the AC-AFPK-IsCT1 peptide drastically sensitized SCC-9 cells to cisplatin, with higher specificity for tumor cells at the tested peptide concentrations (Table 1). The combined treatment of cisplatin with AC-AFPK-IsCT1 at a concentration of 50 µM (corresponding to half the IC50 value of the peptide; Supplemental Figure S2) exhibited great toxicity, resulting in very low cisplatin IC50 values for both 24 h and 48 h exposures (0.4 µM and 0.8 µM, respectively). Lowering the peptide concentrations to 30 µM and 10 µM resulted in cisplatin IC50 values of 19.9 µM and 44.6 µM at 24 h, and 1.1 µM and 1.3 µM at 48 h, respectively, indicating a robust, dose-dependent sensitizing effect of AC-AFPK-IsCT1 on cisplatin-mediated cytotoxicity (Table 1).

Similar results were obtained when using SCC-25 oral squamous cell carcinoma cells. When used as a single agent, the IC50 values for cisplatin were 47.6 µM and 15.2 µM for the 24 h and 48 h treatments, respectively. However, a combination of cisplatin with AC-AFPK-IsCT1 substantially reduced the cisplatin IC50 values. Indeed, as shown in Table 1, association with AC-AFPK-IsCT1 at a concentration of 40 µM (corresponding to half the IC50 value of the peptide for these cells; Supplemental Figure S2) resulted in IC50 values for cisplatin of 24.6 µM and 4.7 µM after 24 h and 48 h of treatment. For peptide concentrations of 20 µM and 10 µM, the cisplatin IC50 values were 31.7 µM and 38.8 µM at 24 h, and 5.1 µM and 6.7 µM at 48 h, respectively.

The cytotoxic potential of cisplatin and its combinations with the AC-AFPK-IsCT1 peptide was next evaluated in normal human fibroblast cells (FN1). As shown in Table 1, the cisplatin IC50 values, when used as a single agent, were 73.11 µM and 3.06 µM at 24 h and 48 h of treatment, respectively. Importantly, when combined with the AC-AFPK-IsCT1 peptide for 24 h, no significant toxicity for any of the tested cisplatin concentrations was observed (Table 1). After 48 h of treatment, some cisplatin-induced cytotoxicity was observed, but the calculated IC50 values were considerably higher than those found for cisplatin used as a single agent (Table 1).

For the J774 macrophage cells, the cisplatin IC50 values were 68.3 µM and 8.7 µM for the 24 h and the 48 h treatments, respectively. As observed with the FN1 cells, a combination of cisplatin with the AC-AFPK-IsCT1 peptide substantially increased the cisplatin IC50 values. As shown in Table 1, for a peptide concentration of 50 µM, the cisplatin IC50 value was 48.57 µM after 48 h of treatment. For peptide concentrations of 30 µM and 10 µM, the cisplatin IC50 values were 42.1 µM and 37.5 µM, respectively, after 48 h of treatment, whereas no noticeable cisplatin-mediated toxicity was detected upon a 24 h treatment (Table 1).

Combined, these findings suggest that the association of cisplatin with the AC-AFPK-IsCT1 peptide maximizes the cisplatin-induced cytotoxicity toward tumor cells while reciprocally reducing the cisplatin-induced toxicity toward normal cells, highlighting the therapeutic potential of this approach.

### 2.2. Impact of the Cisplatin and AC-AFPK-IsCT1 on the Proliferative Activity of Tongue Squamous Cell Carcinoma and Normal Cells

**Figure 1:**
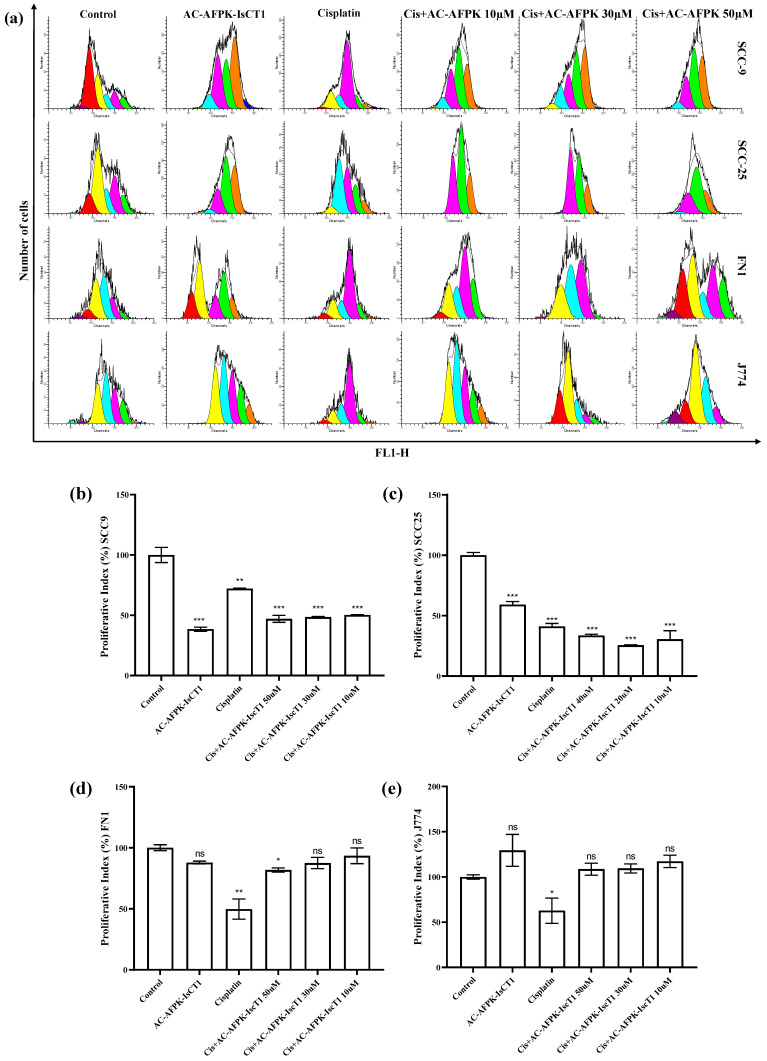
Assessment of the cellular proliferative index was conducted using flow cytometry following a 24 h treatment with cisplatin and AC-AFPK-IsCT1. (**a**) Representative histograms of the proliferative index were analyzed using WinMDI 5.0 software. Each color in the histogram represents a new cell population. (**b**) Proliferative index for SCC-9 tumor cells. (**c**) Proliferative index for SCC-25 tumor cells. (**d**) Proliferative index for normal FN1 cells; (**e**) Proliferative index for normal J774 cells. Bar graphs show the correlation of the effect on proliferative index, expressed as the mean ± SD of three independent experiments. Statistical differences were determined using ANOVA and Tukey–Kramer multiple comparison tests. * *p* < 0.05, ** *p* < 0.01, and *** *p* < 0.001, with “ns” denoting not significant.

We next evaluated the impact of cisplatin, the AC-AFPK-IsCT1 peptide, or their combination on tumor cells’ proliferation. The treatment of SCC-9 cells with AC-AFPK-IsCT1 resulted in a reduction in the proliferative index by 61.1 ± 1.2%, indicating a strong antiproliferative effect (Figure 1b). Cisplatin treatment showed a more moderate reduction of 28.1 ± 0.2%. Interestingly, the combination of cisplatin + AC-AFPK-IsCT1 at concentrations of 50 µM, 30 µM, and 10 µM further decreased the proliferative index by 51.2 ± 0.9%, 52.3 ± 0.4%, and 50.4 ± 0.5%, respectively (Figure 1b).

AC-AFPK-IsCT1 treatment of SCC-25 cells reduced the tumor cells’ proliferative index by 40.7 ± 0.3%, indicating a moderate antiproliferative effect (Figure 1c). Cisplatin resulted in a stronger reduction of 57.4 ± 0.3%. Here again, the combination of cisplatin + AC-AFPK-IsCT1 at concentrations of 40 µM, 20 µM, and 10 µM further decreased the proliferative index by 66.6 ± 0.1%, 73.3 ± 0.02%, and 72.3 ± 0.5%, respectively (Figure 1c).

Cisplatin treatment of normal FN1 cells significantly reduced the proliferative index by 50.5 ± 0.8% compared to the control (Figure 1d). Strikingly, the combination of cisplatin + AC-AFPK-IsCT1 used at 10 µM restored the proliferative index to 91.2 ± 0.1% of the control cells, indicating a partial reversal of the antiproliferative effect observed with cisplatin used as a single agent (Figure 1d). The other treatments, including AC-AFPK-IsCT1 used alone and in the remaining combinations, did not induce significant changes in the proliferative index, suggesting a minimal impact on the cell proliferation in FN1 cells.

For the normal J774 cells, cisplatin treatment resulted in a significant reduction in the proliferative index, decreasing it by 47.5 ± 0.35% compared to the control cells, similar to the findings observed in the FN1 cells (Figure 1e). The other treatment conditions, including AC-AFPK-IsCT1 and the combination therapies, did not induce significant changes in the proliferative index, suggesting that these treatments had a minimal impact on the cell proliferation in J774 cells (Figure 1e).

### 2.3. Synergistic Interaction of Cisplatin and AC-AFPK-IsCT1 in SCC-9 and SCC-25 Cells

**Figure 2:**
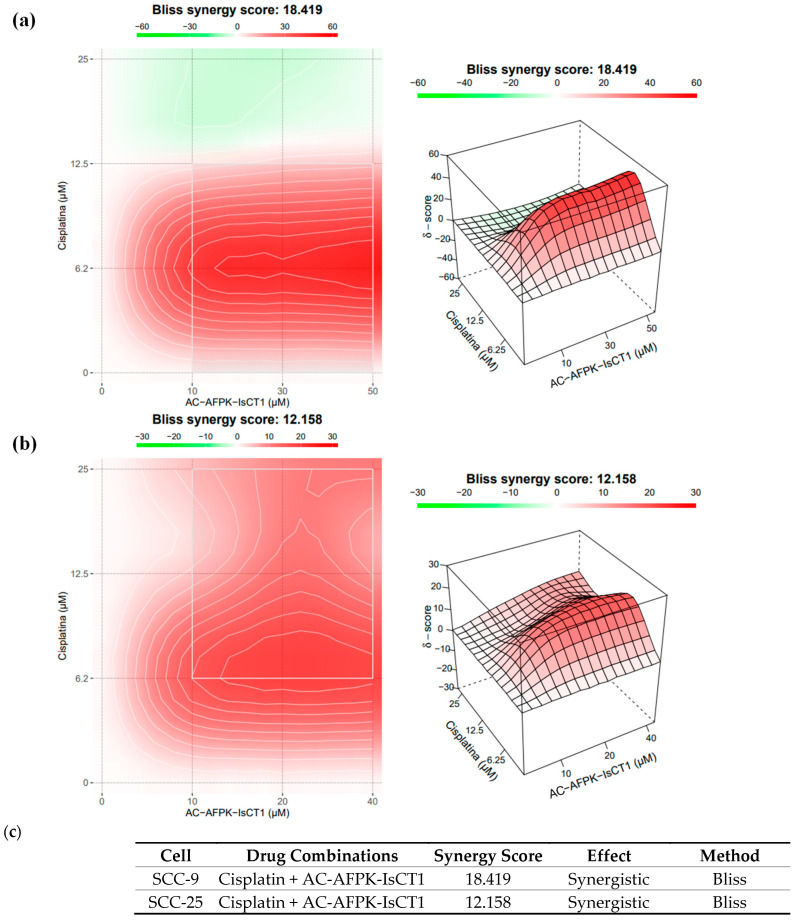
Evaluation of the synergistic interaction between cisplatin and AC-AFPK-IsCT1 in SCC-25 tumor cells using the Bliss model. (**a**) Pharmacological effect of the association of cisplatin with the AC-AFPK-IsCT1 peptide in SCC-9 tumor cells. (**b**) Pharmacological effect of the association of cisplatin with the AC-AFPK-IsCT1 peptide in SCC-25 tumor cells. (**c**) Table presenting the exact values of synergy scores and the corresponding effects for each concentration combination. Heatmap shows regions of antagonism (green, ≤0), additivity (white to light red, >0 to <10), and synergy (dark red, ≥10) for varying concentrations of cisplatin and AC-AFPK-IsCT1. Color intensity indicates the magnitude of synergy or antagonism; 3D plot displaying the Bliss synergy score.

We next quantitatively investigated the synergistic therapeutic effect of the combination of cisplatin and AC-AFPK-IsCT1. As shown in Figure 2a, a Bliss synergy score of 18.419 indicated a moderately strong synergistic effect between cisplatin and AC-AFPK-IsCT1 for SCC-9 cells. This suggests that the combination of these two agents could enhance the therapeutic efficacy of cisplatin used at lower doses, potentially reducing the side effects associated with higher doses of cisplatin. The most pronounced synergy was observed with concentrations of AC-AFPK-IsCT1 around 25-50 µM, highlighting the peptide role in enhancing the cytotoxic effect of cisplatin on SCC-9 cells (Figure 2a).

This synergy may also suggest a potential clinical application for this drug combination, where it could be used to achieve better outcomes in the treatment of squamous cell carcinoma by targeting cancer cells more effectively while minimizing the toxicity to normal cells.

The same analysis was then performed in SCC-25 cells. A Bliss synergy score of 12.158 indicates that, although there is a synergistic interaction between cisplatin and AC-AFPK-IsCT1 in SCC-25 cells, this effect is less pronounced than what is observed in SCC-9 cells. This suggests that while the combination remains effective, SCC-25 cells may require different dosing strategies or might exhibit slight resistance to the combination at certain concentrations (Figure 2b). The observed synergy at mid-range concentrations could still be therapeutically beneficial, allowing for potential dose reduction in cisplatin, thereby lowering the toxicity while maintaining the efficacy in tumor cells. However, the lower overall score compared to SCC-9 indicates that SCC-25 cells may not respond as robustly to this combination, suggesting that further investigation may be required to optimize the combination’s efficacy in this cellular context.

### 2.4. Cell Cycle Modulation and DNA Fragmentation

**Figure 3:**
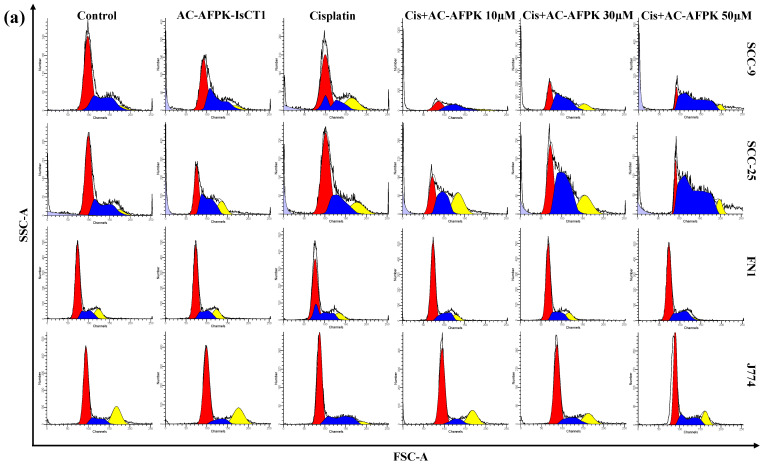

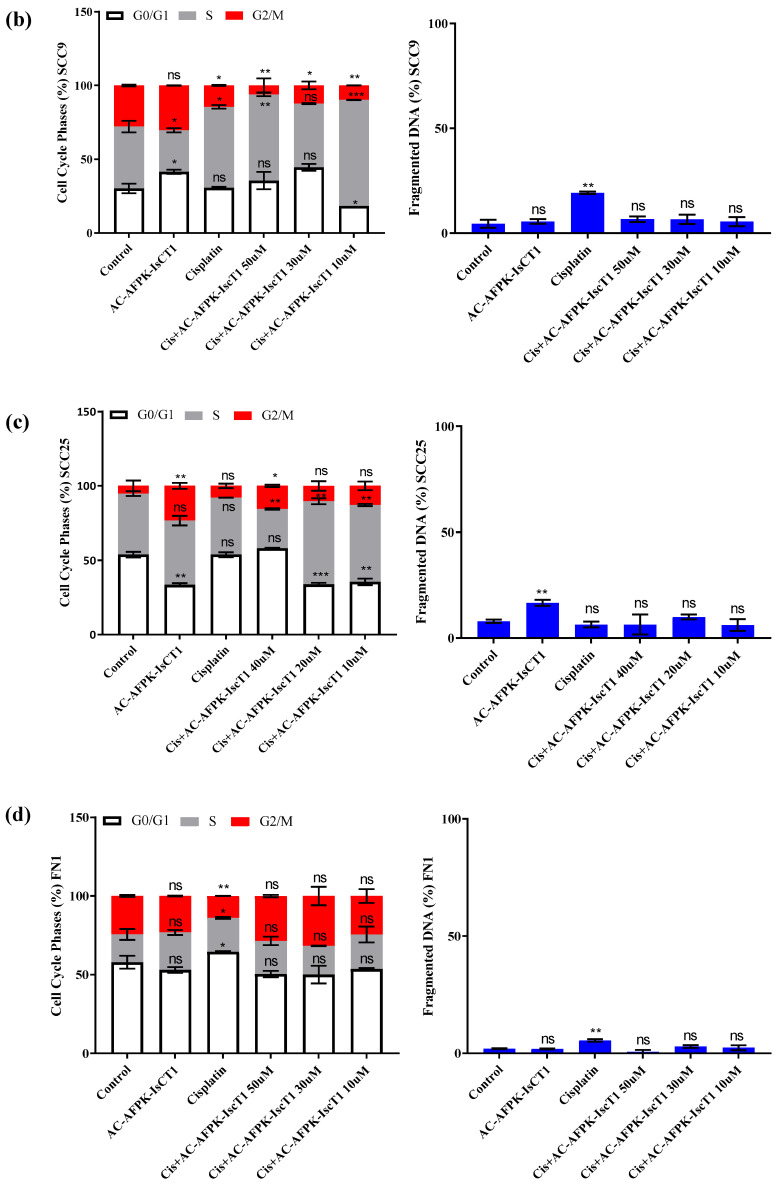

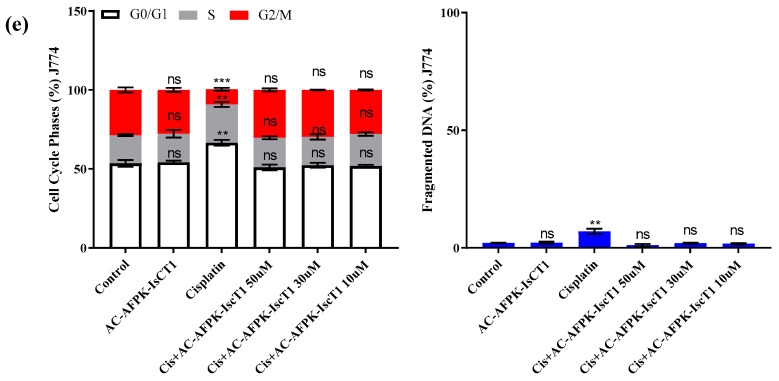
Analysis of cell cycle phases and fragmented DNA after 24 h treatment with cisplatin, AC-AFPK-IsCT1 peptides and cisplatin + AC-AFPK-IsCT1 combination. (**a**) Representative histograms of cell distribution across cell cycle phases, with color coding as follows: light blue (bottom left corner) indicates fragmented DNA, red color indicates cells in G0/G1 phase, blue color indicates cells in S phase and yellow color indicates cells in G2/M phase; (**b**) SCC-9 tumor cells; (**c**) SCC-25 tumor cells; (**d**) FN1 normal cells; (**e**) J774 normal cells. Bar graph showing the correlation of the effect on cell cycle and fragmented DNA, expressed as the mean ± SD of three independent experiments. Statistical differences were obtained using ANOVA and Tukey–Kramer multiple comparison tests. * *p* < 0.05, ** *p* < 0.01, and *** *p* < 0.001 denote significance. ns = not significant.

We then analyzed the impact of an AC-AFPK-IsCT1 peptide–cisplatin combination on the cell cycle of tongue squamous cell carcinoma cells or normal cells. The treatment of SCC-9 cells with AC-AFPK-IsCT1 or cisplatin induced distinct cell cycle effects. As shown in Figure 3b, the treatment of SSC-9 cells with AC-AFPK-IsCT1 led to a cell cycle arrest in the G0/G1 phase (40.43 ± 2.1%), while cisplatin treatment caused a significant accumulation of cells in the S phase (55.58 ± 3.4%). The combination of cisplatin + AC-AFPK-IsCT1 at 50 µM resulted in the highest S-phase arrest (57.60 ± 1.9%) and a significant reduction in the G2/M phase (2.77 ± 3.0%) (Figure 3b). Lower concentrations, such as 30 µM, increased the G0/G1 phase (46.10 ± 2.8%), while 10 µM caused a substantial S-phase accumulation (71.99 ± 2.4%) (Figure 3b). In terms of DNA fragmentation, cisplatin induced the highest fragmentation (19.60 ± 1.6%), while the combination treatments, particularly at 50 µM and 30 µM of the AC-AFPK-IsCT1 peptide, induced moderate fragmentation (7.56 ± 2.1% and 5.01 ± 2.7%, respectively) (Figure 3b).

The cell cycle analysis in SCC-25 cells treated with AC-AFPK-IsCT1, cisplatin, and their combinations revealed distinct effects. AC-AFPK-IsCT1 reduced the G0/G1 phase (32.73 ± 2.2%) and increased the G2/M phase (21.88 ± 1.5%), indicating G2/M arrest. Cisplatin primarily induced G0/G1 arrest (54.97 ± 3.4%) and lower S-phase accumulation (38.18 ± 2.7%) (Figure 3c). The combination of cisplatin + AC-AFPK-IsCT1 (40 µM) further increased G0/G1 arrest (58.35 ± 1.3%) and decreased the S phase (26.62 ± 1.9%). At 20 µM, the combination shifted cells towards S-phase accumulation (54.34 ± 2.1%), while 10 µM showed a balance between the G0/G1 (33.92 ± 2.4%) and S phases (51.09 ± 1.7%). In terms of DNA fragmentation, AC-AFPK-IsCT1 caused significant fragmentation (17.59 ± 1.5%), while cisplatin resulted in minimal fragmentation (5.41 ± 2.2%). Combination treatments showed moderate fragmentation, especially at 20 µM (9.14 ± 2.6%) and 40 µM (3.11 ± 2.1%) of AC-AFPK-IsCT1 (Figure 3c).

In both the FN1 and J774 normal cells, cisplatin was the only treatment that induced statistically significant changes. Cisplatin led to a notable increase in DNA fragmentation, averaging 5.52 ± 0.8% in the FN1 cells (Figure 3d) and 7.03 ± 1.1% in the J774 cells (Figure 3e), compared to control groups. Additionally, cisplatin caused a significant accumulation of cells in the G0/G1 phase, with averages of 64.51 ± 1.6% in the FN1 cells and 66.54 ± 1.9% in the J774 cells, alongside a reduction in the G2/M phase (averaging 13.93 ± 0.9% in the FN1 cells and 9.64 ± 1.2% in the J774 cells) (Figure 3d,e). Importantly, none of the other treatments, including AC-AFPK-IsCT1 and its combinations with cisplatin, induced significant alterations in either DNA fragmentation or cell cycle distribution in either cell line. This suggests that the effects of cisplatin are selective, primarily impacting the normal FN1 and J774 cells and that the AC-AFPK-IsCT1 peptide can abolish cisplatin-mediated alteration of the cellular cycle on these non-tumor cells. This highlights the cytotoxicity of cisplatin in normal cells, which may raise concerns about its toxicity in non-cancerous tissues.

### 2.5. Impact of Cisplatin and AC-AFPK-IsCT1 on the Mitochondrial Membrane Electrical Potential (ΔΨm)

**Figure 4:**
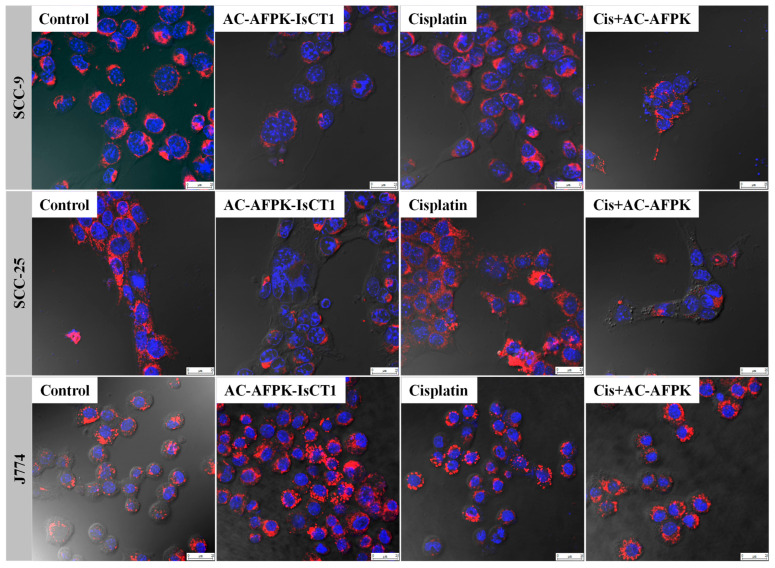
Photomicrographs of tongue squamous cell carcinoma tumor cells (SCC-9 and SCC-25) and normal macrophages (J774), with mitochondria stained red (MitoRED) and nuclei stained blue (DAPI), analyzed using confocal laser microscopy. Analysis after 24 h treatment with cisplatin, AC-AFPK-IsCT1 peptides, and cisplatin + AC-AFPK-IsCT1 combination.

We next examined the effects of cisplatin and AC-AFPK-IsCT1 on the mitochondrial membrane potential (ΔΨm), an important factor in mitochondrial health. The qualitative microscopy image analysis of SCC-9 cells, based on the fluorescence intensity and presence or absence of MitoRED labeling, demonstrated significant changes in mitochondrial morphology and potential following the various treatments. As shown in Figure 4, mitochondria displayed robust and dense MitoRED staining in the control group, indicative of a stable mitochondrial membrane potential (ΔΨm). Treatment with AC-AFPK-IsCT1 resulted in notable mitochondrial fragmentation and a visible decrease in MitoRED staining intensity, suggesting signal attenuation compatible with reduced ΔΨm and mitochondrial degradation (Figure 4). Cisplatin treatment caused moderate mitochondrial fragmentation and slightly reduced MitoRED staining, indicating ΔΨm loss. The combination of cisplatin + AC-AFPK-IsCT1 led to severe mitochondrial fragmentation and sparse MitoRED staining, along with pronounced nuclear condensation, suggesting, from a fluorescence-based perspective, advanced mitochondrial dysfunction and potential apoptosis (Figure 4).

As shown in Figure 4, whereas the control SCC-25 cells exhibited elongated and interconnected mitochondria with vibrant red staining, AC-AFPK-IsCT1 treatment resulted in mitochondrial fragmentation and highly reduced MitoRED staining, highlighting a disruption in ΔΨm as visually inferred from the staining patterns. Cisplatin treatment induced moderate mitochondrial fragmentation with substantially sparse red staining, reflecting a significant loss of ΔΨm and indications of apoptosis (Figure 4). However, the combination of cisplatin + AC-AFPK-IsCT1 in SCC-25 cells led to pronounced mitochondrial disruption and nuclear condensation, along with very faint red staining, which visually supports the interpretation of mitochondrial depolarization and apoptotic progression (Figure 4).

Interestingly, AC-AFPK-IsCT1 treatment of normal J774 cells markedly increased the MitoRED staining intensity compared to the control, suggesting a significant rise in mitochondrial activity and potential cellular robustness against the treatment (Figure 4). Cisplatin treatment in J774 cells led to slight mitochondrial disruption, characterized by smaller mitochondria with reduced MitoRED staining, pointing to mitochondrial depolarization and damage under qualitative fluorescence evaluation (Figure 4). However, the combination of cisplatin and AC-AFPK-IsCT1 showed an interesting pattern of mitochondrial clustering and an increase in MitoRED staining, implying, from a morphological and staining standpoint, a partial restoration of ΔΨm and improved mitochondrial function relative to cisplatin treatment alone, further pointing towards a beneficial role of AC-AFPK-IsCT1 (Figure 4).

Combined, these data confirm the antitumoral properties of the AC-AFPK-IsCT1 and cisplatin combination therapy while highlighting the protective role of AC-AFPK-IsCT1 for cisplatin-induced cytotoxicity in non-cancerous cells.

### 2.6. Effect of Cisplatin and AC-AFPK-IsCT1 Peptide on the Expression of Proteins Involved in Cell Death and Proliferation in Tumor Cells

**Figure 5:**
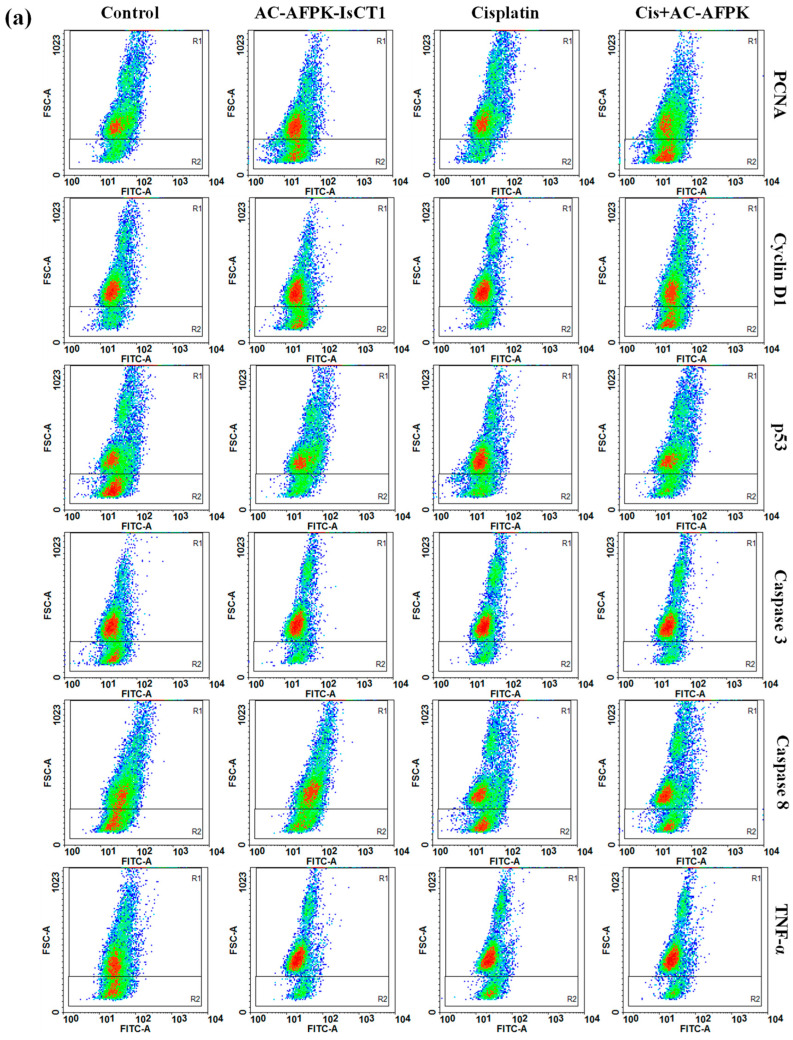

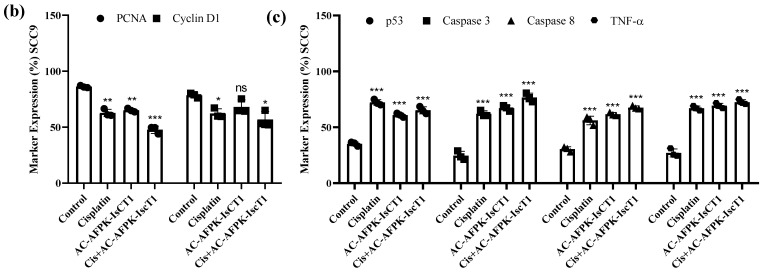
Analysis of marker expression in tongue squamous cell carcinoma tumor cells (SCC-9). The expression of markers was quantified using flow cytometry after 24 h treatment with cisplatin, AC-AFPK-IsCT1 peptides, and cisplatin + AC-AFPK-IsCT1 combination. (**a**) Representative density plots display the distribution of cells according to fluorescence intensity, with color gradients indicating event density: red for high-density cell populations, green for intermediate density, and blue for low-density or sparse cell distributions; (**b**) marker expression for PCNA and Cyclin D1; (**c**) marker expression for p53, caspase-3 and -8, and TNF-α bar graphs present protein expression levels as the mean ± SD from three independent experiments. Statistical differences were determined using ANOVA and Tukey–Kramer multiple comparison tests. * *p* < 0.05, ** p < 0.01, and *** *p* < 0.001 denote significance. ns = not significant.

We then investigated the possible mechanisms involved in the anti-cancer effect of cisplatin and AC-AFPK-IsCT1 in oral squamous cell carcinoma cells. For that purpose, the expression of cell death and cell cycle progression-related proteins was evaluated prior to and after tumor cells’ treatments with the drugs. As shown in Figure 5b, a 24 h treatment of SCC-9 cells with cisplatin significantly reduced the PCNA (62.61 ± 3.11% vs. 86.14 ± 0.99% in the control cells) and Cyclin D1 (62.81 ± 4.10% vs. 78.38 ± 2.15% in the control cells) levels, indicating a decrease in cell proliferation and cell cycle progression markers. AC-AFPK-IsCT1 monotherapy treatment led to a moderate reduction in PCNA expression (65.13 ± 1.59%) with a minimal effect on Cyclin D1 (71.35 ± 5.02%) (Figure 5b). Interestingly, the combination of cisplatin + AC-AFPK-IsCT1 had the most pronounced effect, reducing PCNA expression to 47.04 ± 3.53% and Cyclin D1 to 56.11 ± 4.66%, suggesting the strong inhibition of proliferation and cell cycle progression (Figure 5b).

Cisplatin treatment of SSC-9 cells also significantly increased p53 expression (72.37 ± 2.11% vs. 21.83 ± 3.9% in the control cells), and both caspase-3 and caspase-8 expressing cells rose to 61.18 ± 2.54% and 55.40 ± 2.80%, respectively, indicating the activation of apoptotic pathways (Figure 5b). TNF-α expression also increased to 66.57 ± 2.79% vs. 20.45 ± 2.8% in the control cells, suggesting enhanced pro-inflammatory signaling upon cisplatin exposure (Figure 5b). In contrast, AC-AFPK-IsCT1 treatment only resulted in moderate increases in p53 (60.13 ± 1.73%), caspase-3 (67.41 ± 2.18%), and caspase-8 (61.00 ± 1.99%) expressing cells, with a higher rise in TNF-α (69.58 ± 2.77%) expression, indicating a more controlled apoptotic and inflammatory response compared to cisplatin used as a single agent (Figure 5b). As expected from our previous results, the combination of cisplatin + AC-AFPK-IsCT1 further elevated the p53 levels (65.43 ± 2.24%) and significantly boosted caspase-3 (76.48 ± 1.89%) and caspase-8 (67.09 ± 1.73%) expression, indicating strong apoptotic induction. The TNF-α levels were also higher (72.50 ± 2.18%), suggesting a synergistic effect in apoptosis induction when the drugs are combined (Figure 5b).

**Figure 6:**
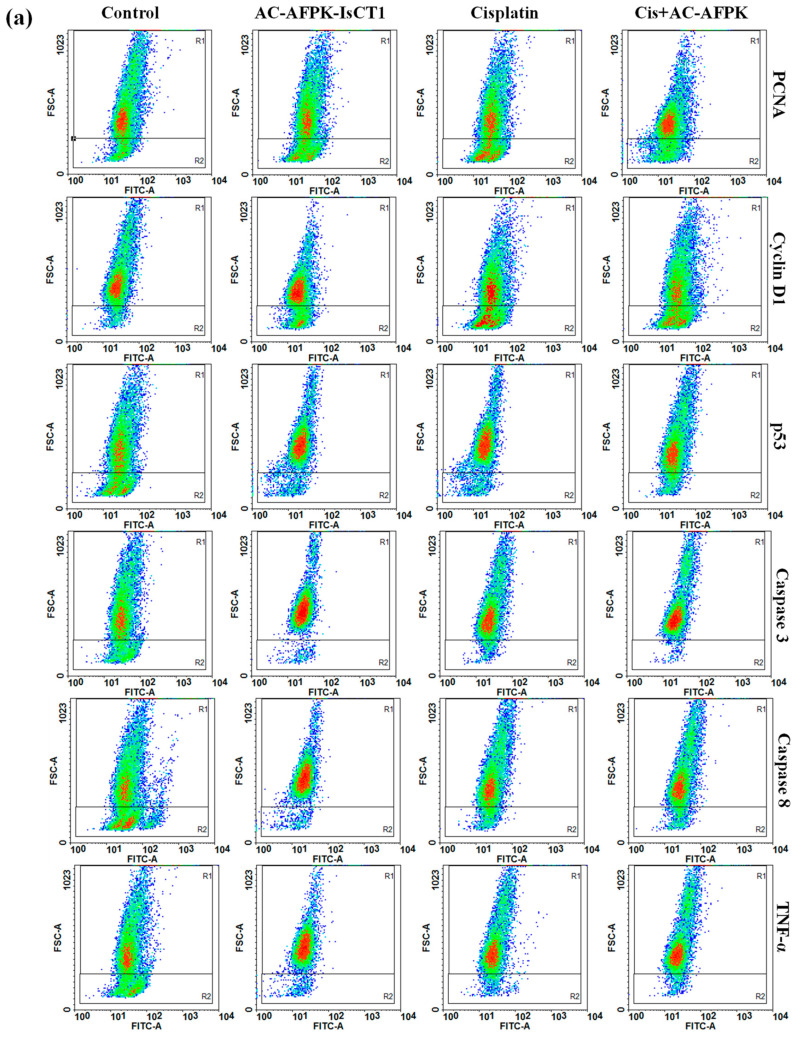

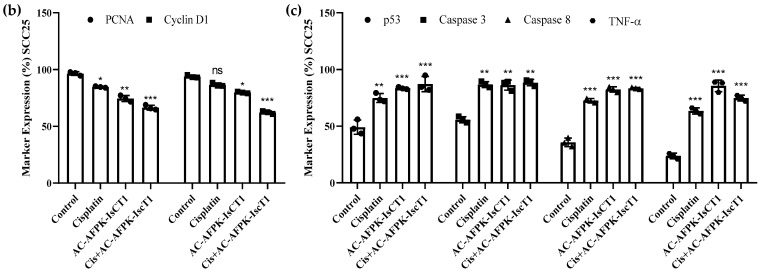
Analysis of marker expression in tongue squamous cell carcinoma tumor cells (SCC-25). The expression of markers was quantified using flow cytometry after 24 h treatment with cisplatin, AC-AFPK-IsCT1 peptides, and cisplatin + AC-AFPK-IsCT1 combination. (**a**) Representative density plots display the distribution of cells according to fluorescence intensity, with color gradients indicating event density: red for high-density cell populations, green for intermediate density, and blue for low-density or sparse cell distributions; (**b**) marker expression for PCNA and Cyclin D1; (**c**) marker expression for p53, caspase-3 and -8, and TNF-α bar graphs present protein expression levels as the mean ± SD from three independent experiments. Statistical differences were determined using ANOVA and Tukey–Kramer multiple comparison tests. * *p* < 0.05, ** p < 0.01 and *** *p* < 0.001 denote significance. ns = not significant.

We then assessed the expression of PCNA and Cyclin D1 in SCC-25 cells following 24 h of treatment with cisplatin, AC-AFPK-IsCT1, and their combination. Cisplatin treatment led to a decrease in PCNA expression to 84.65 ± 0.65% expressing cells vs. 96.41 ± 1.1% in the control cells, as well as in Cyclin D1 expression, which was reduced to 86.62 ± 1.37% vs. 93.83 ± 1.3% in the control cells, indicating an inhibition of cell proliferation and cell cycle progression (Figure 6b). Contrary to what was observed in SSC-9 cells, the treatment of SSC-25 cells with AC-AFPK-IsCT1 caused a more pronounced reduction in PCNA expression (74.43 ± 2.26%) compared to the control group, with Cyclin D1 expression also decreased to 79.71 ± 0.37%, suggesting a more potent effect on cell cycle regulation (Figure 6b). Here again, the combination of cisplatin + AC-AFPK-IsCT1 resulted in the most significant decrease in PCNA expression (66.47 ± 2.06%), alongside a marked reduction in Cyclin D1 (62.92 ± 0.99%), indicating a strong inhibition of proliferation and cell cycle activity (Figure 6b).

Cisplatin treatment of SSC-25 cells significantly increased the p53 expression to 75.29 ± 3.67% vs. 25.77 ± 2.0% in the control cells, with a substantial rise in both caspase-3 (86.61 ± 2.33% vs. 15.25 ± 2.9% in the control cells) and caspase-8 (72.60 ± 1.67% vs. 29.02 ± 1.7% in the control cells), indicating strong apoptotic activation (Figure 6c). TNF-α expression also increased to 63.42 ± 1.96% vs. 20.30 ± 3.2% in the control cells, suggesting a marked inflammatory response. AC-AFPK-IsCT1 treatment led to a further increase in p53 levels (83.27 ± 0.95%), along with enhanced caspase-3 (86.87 ± 4.55%) and caspase-8 (82.07 ± 2.91%), indicating a strong induction of apoptosis (Figure 6c). TNF-α expression also increased significantly upon treatment with the combination (84.94 ± 4.66%), indicating a heightened inflammatory response compared to cisplatin used alone (Figure 6c). As witnessed for SSC-9 cells, the combination of cisplatin + AC-AFPK-IsCT1 resulted in the highest p53 expression (87.79 ± 3.46%), with strong activation of caspase-3 (88.39 ± 2.36%) and caspase-8 (83.72 ± 2.13%), indicating maximal apoptotic activity. TNF-α expression reached its peak at 74.11 ± 3.29%, further suggesting enhanced pro-inflammatory signaling (Figure 6c).

## 3. Discussion

Cisplatin is a highly toxic drug impacting both cancerous and noncancerous cells, which can limit its use in clinics [8,9]. It is therefore of importance to develop new therapeutic strategies that can reduce cisplatin indiscriminate action without diminishing its anti-tumor efficacy. In this study, we demonstrate that the combination of cisplatin and the recently developed AC-AFPK-IsCT1 peptide strongly enhances cisplatin cytotoxicity toward tumor cells while reducing the adverse side effects of cisplatin on normal cells, highlighting the therapeutic potential of this approach in the context of oral squamous cell carcinoma. Additionally, the substantial reduction in proliferative activity observed in tumoral cells with combination treatments underscores the interest of testing this approach in targeting highly proliferative tumors while minimizing cisplatin off-target toxicity.

Cisplatin remains one of the most widely used chemotherapeutic agents for various types of cancer, particularly head and neck squamous cell carcinoma [15,16]. However, its therapeutic efficacy is often limited by its systemic toxicity and the development of resistance [17]. Recent research has focused on combination therapies to overcome these limitations [18,19]. Peptide-based therapies, such as those involving the AC-AFPK-IsCT1 peptide, have shown potential in enhancing the efficacy of cisplatin by targeting the specific pathways involved in cancer cell survival, apoptosis, and proliferation [15]. Understanding the molecular mechanisms activated by these combinations is crucial for optimizing the treatment strategies and minimizing the side effects [15].

Several studies suggest that the enhanced cytotoxicity observed in combination therapies involving cisplatin and various peptides is mediated by the activation of apoptotic pathways [18]. Cisplatin is known to induce DNA damage and trigger the mitochondrial (intrinsic) apoptotic pathway by activating p53, a key regulator of cell cycle arrest and apoptosis. The activation of p53 leads to the upregulation of pro-apoptotic proteins, including Bax and Bak, and downregulation of anti-apoptotic proteins like Bcl-2, resulting in mitochondrial outer membrane permeabilization (MOMP) and the release of cytochrome c, which subsequently activates caspase-9 and caspase-3 [15,17,20,21]. Studies have shown that peptides, including antimicrobial peptides like IsCT1 derivatives, can synergistically enhance cisplatin-induced apoptosis by promoting mitochondrial dysfunction and facilitating the activation of caspases [22].

The AC-AFPK-IsCT1 peptide may exert its antitumor effects through the disruption of the mitochondrial membrane potential (ΔΨm), as observed in previous studies [23]. The mitochondrial dysfunction observed in peptide-treated cancer cells suggests that AC-AFPK-IsCT1 directly affects the mitochondria, promoting depolarization and apoptosis [13,23,24]. This is in line with studies demonstrating that peptides can act as mitochondrial-targeting agents, directly altering ΔΨm and leading to increased reactive oxygen species (ROS) production and oxidative stress, which further amplify cisplatin-induced DNA damage and apoptosis [25,26]. In this respect, our results show that the treatment of SSC cells with AC-AFPK-IsCT1 induces a loss of ΔΨm and early mitochondria dysfunction.

The combination of cisplatin with peptides may engage additional cell death pathways, including the extrinsic apoptotic pathway, which is mediated by death receptors such as Fas and the TNF-related apoptosis-inducing ligand (TRAIL) [27,28]. The activation of these receptors leads to the recruitment of the Fas-associated death domain (FADD) and the activation of caspase-8, which can directly activate caspase-3 or cleave Bid, a pro-apoptotic Bcl-2 family member, to induce mitochondrial damage and amplify the apoptotic signal [29,30]. The observed upregulation of caspase-8 in AC-AFPK-IsCT1–cisplatin-treated cells suggests that the extrinsic pathway may also be involved in the observed cytotoxicity, which might be of interest in tumor cells resistant to intrinsic pathway activation alone [31].

In addition to apoptosis, cell cycle arrest plays a significant role in the therapeutic efficacy of peptide–cisplatin combinations. Cisplatin typically induces cell cycle arrest in the S phase by interfering with DNA replication through the formation of inter- and intra-strand crosslinks [15]. Peptides, however, may induce cell cycle arrest in different phases, such as G0/G1, as shown in the studies on antimicrobial peptides [23,32,33]. This differential effect on the cell cycle may enhance the cytotoxicity of cisplatin by preventing cancer cells from escaping apoptosis through cell cycle progression [26]. The observed G2/M arrest with the AC-AFPK-IsCT1 peptide treatment in combination with cisplatin indicates a blockade in the mitotic phase, which further sensitizes tumor cells to DNA damage-induced apoptosis [34].

Furthermore, the anti-proliferative effects of AC-AFPK-IsCT1–cisplatin combinations are also likely influenced by the inhibition of the key proteins involved in cell cycle progression, such as PCNA and Cyclin D1. PCNA acts as a processivity factor for DNA polymerase and is crucial for DNA replication and repair, while Cyclin D1 regulates the transition from the G1 to the S phase of the cell cycle [35,36]. The downregulation of these proteins in AC-AFPK-IsCT1-treated cells suggests that these combinations inhibit the proliferative capacity of cancer cells by disrupting DNA synthesis and cell cycle control, making them more susceptible to apoptosis [37].

Overall, the combination of cisplatin and peptides such as AC-AFPK-IsCT1 holds significant promise for enhancing the therapeutic efficacy of cisplatin by engaging multiple molecular pathways. By promoting mitochondrial dysfunction, activating both intrinsic and extrinsic apoptotic pathways, inducing cell cycle arrest, and inhibiting the key proliferation markers, these combinations offer a multifaceted approach to overcoming cisplatin resistance while reducing toxicity. Future studies should focus on further elucidating these mechanisms and exploring the potential of such combinations in clinical settings, especially in cancer types where cisplatin resistance is prevalent.

## 4. Materials and Methods

### 4.1. Solid-Phase Peptide Synthesis (SPPS), Purification, and Analysis

The peptide, AC-AFPK-IscT1 (COOH-ALGKFWPKIKSLF-CONH2), was synthesized using a peptide synthesizer (PS3-SyncTechnologies) employing the solid-phase synthesis methodology and the fluoromethylcarbonil (Fmoc) strategy [23]. Purification assays were conducted through semi-preparative reverse-phase high-performance liquid chromatography (RP-HPLC) on a Delta Prep 600 (Waters Associates, Milford, MA, USA). Selected fractions containing the purified peptides were collected and lyophilized. The purity was assessed using an Alliance HPLC system (Waters Associates) (Figure S1) and characterized using liquid chromatography/electrospray ionization mass spectrometry (LC/ESI-MS) with a 6130 Infinity mass spectrometer coupled to a 1260 HPLC system (Agilent, Santa Clara, CA, USA) (Figure S1).

### 4.2. Cell Culture

The cell lines used for oral cavity squamous carcinoma, SSC-9 (ATCC^®^ CRL-1629™), SCC-25 (ATCC^®^ CCRL-1628™), and J-774 (ATCC^®^ TIB-67™), were obtained from the American Type Culture Collection (Manassas, VA, USA). The normal human fibroblast cell line, FN1 (CAPPesq HCFMUSP No. 921/06), was isolated by Professor Dr. Durvanei Augusto Maria. The SCC-9 and SCC-25 cells were cultured in a 1:1 mixture of DMEM (Cultilab, Sao Paulo, Brazil) and Ham’s F12 medium (Cultilab, Sao Paulo, Brazil), supplemented with 1.2 g/L sodium bicarbonate, 2.5 mM L-glutamine, 15 mM HEPES, 0.5 mM sodium pyruvate, 400 ng/mL hydrocortisone, and 10% fetal bovine serum. The FN1 cells were cultured in RPMI-1640 medium (LGC Biotecnologia, Sao Paulo, Brazil) containing 10% fetal bovine serum, 100 units/mL Penicillin G, and 100 μg/mL streptomycin. Cells were incubated at 37 °C with 5% CO_2_.

### 4.3. Cytotoxic Activity Determination Using the MTT Colorimetric Method

Tumor cells (SSC-9 and SSC-25) and normal cells (FN1 and J-774) were incubated in 96-well plates at a density of 10^5^ cells/mL for 24 h. Subsequently, treatments were administered as described in Table 2 for 24 and 48 h. After the treatment period, the supernatant was aspirated, and 100 μL of MTT at a concentration of 5 mg/mL (Calbiochem, Darmstadt, Germany) was added to the plate and incubated for 3 h at 37 °C with 5% CO_2_. Afterward, the content was removed, and 200 μL of methanol was added to dissolve the formazan crystals. The absorbance was then measured at a wavelength of 540 nm using a microplate reader.

### 4.4. CFSE-DA Proliferation Assay

The tumor cells (SSC-9 and SSC-25) and normal cells (FN1 and J-774) were incubated in 24-well plates at a density of 10^5^ cells/mL for 24 h. The treated and control groups were incubated with the carboxyfluorescein marker (CFSE-DA Thermo Fisher, Waltham, MA, USA, KITC34571). CFSE-DA was diluted in 0.1% PBS human albumin and added to the cell-containing medium. After 24 h of treatment, the cells were trypsinized, transferred to a conical tube, and centrifuged at 1500 rpm for 5 min. The supernatant was discarded, and the pellet was resuspended in 1 mL of 4% paraformaldehyde for 30 min. The cells were then centrifuged, the supernatant was discarded, and the pellet was resuspended in 200 μL of FACS buffer. Readings were performed on a FACScanto flow cytometer (BD, Franklin Lakes, NJ, USA) with the number of events set at 10,000 events, and histograms were acquired and analyzed using ModFit LT 5.0 software.

### 4.5. Cell Cycle Phase and DNA Fragmentation Analysis via Flow Cytometry

The tumor cells (SSC-9 and SSC-25) and normal cells (FN1 and J-774) were subjected to treatment for 24 h at the IC50 concentration. The treated and control cells were trypsinized and centrifuged at 1200 rpm for 5 min. The resulting pellet was resuspended in 1 mL of cold buffer, followed by the addition of 3 mL of absolute ethanol. The samples were stored at −20 °C for 24 h. The samples were then centrifuged at 1500 rpm for 10 min and resuspended in 200 μL of FACS buffer containing 0.1% Triton X-100 (Sigma-Aldrich, St. Louis, MO, USA), 50 μg/mL propidium iodide (Sigma-Aldrich), and 1 µL of RNAse (200×). This mixture was kept at room temperature, protected from light, for 30 min. The samples were then transferred to cytometry tubes and analyzed using a FACScanto flow cytometer (BD) with 10,000 events, and histograms were acquired and analyzed using ModFit LT 5.0 software.

### 4.6. Morphological Evaluation via Laser Confocal Microscopy

The tumor and normal cells were grown in 24-well plates containing round coverslips with, respectively, RPMI and Leibovitz medium supplemented with 10% FBS and incubated with 5% CO_2_ at 37 °C for 24 h. The samples from the control and treated groups were subjected to the culture media removal process and washed with RPMI medium. A total of 200 nML−1 of MitoRed (Sigma-Aldrich, USA) was then added, and the cells were incubated for 1 h in the dark at 37 °C. Following incubation, the cells were washed with PBS, incubated with MitoRed, and fixed with 4% paraformaldehyde for 30 min. The cells were then washed with PBS and incubated. A total of 100 µL of phalloidin (Sigma-Aldrich, USA) was added, and the cells were placed, for 1 h, in the dark at room temperature. The excess phalloidin was removed and the cells were washed with a culture medium. Coverslips were placed on the slides for observation using the Confocal Laser fluorescence microscope (Fluoview™ 300, Olympus Corporation, Tokyo, Japan), and the images were documented and analyzed.

### 4.7. Evaluation of Cellular Marker Expression via Flow Cytometry

The tumor cells, SSC-9 and SSC-25, and both the treated and control groups (10^5^ cells/mL) were incubated at 4 °C for 1 h with 1 μg of specific antibodies conjugated with Alexa Fluor™ 488 (Thermo Fisher Scientific, Waltham, MA, USA). Various markers associated with cell death and cell cycle progression regulators, such as caspase-3 and -8, cyclin D1, PCNA, p53, and TNF-α conjugated with Alexa Fluor™ 488, were used for the SCC-9 and SCC-25 cells. For intracellular markers, TrionX 0.1% was used for 30 min at room temperature before antibody labeling. Following centrifugation at 1500 rpm and washing with PBS, the supernatant was discarded, and the pellet was resuspended in 200 μL of FACS buffer. The reading and analysis of receptor expression on the cell surface of the tumor cells were carried out using a FACScanto flow cytometer (BD) with the fluorescence intensity of FL1-H for the number of events (10,000 events), and the DotPlots were acquired and analyzed using FCS Express 6 software.

### 4.8. SynergyFinder 2.0 Analysis of Multiple Drug Combinations

To determine the potential synergy of the drug, a matrix study was performed with the AC-AFPK-IscT1 peptide and cisplatin. The combination matrix was tested on two cell lines: SCC-9 and SCC-25. SynergyFinder 2.0 software quantified the degree of synergy as the excess over the multiplicative effect of single drugs as if they acted independently (Bliss), and the following higher-order formulations were used to quantify the drug combination (S) synergy for the multiple drug combination effect measured between 2 drugs:

$$S_{BLISS}=E_{A,B}-\left(E_{A}+E_{B}\right)\;Synergy\;Score=\frac{-10g(p)}{\log(0.05)}\;\times \;\frac{t}{\left|t\right|}\;$$

### 4.9. Statistical Analyses

All the values obtained from the different cell lines are expressed as mean ± standard deviation. After obtaining the individual values for each treated and control cell line, the results were tabulated and analyzed using GraphPad Prism 5.0 and 8.0 software. Data analysis was performed by comparing three or more groups with non-parametric distribution using analysis of variance (ANOVA), followed by the Tukey–Kramer multiple comparison test, considering *p* < 0.05 as the critical level for significance.

## 5. Conclusions

In conclusion, the combination of cisplatin with the synthetic peptide AC-AFPK-IsCT1 significantly enhances cisplatin cytotoxicity against squamous cell carcinoma cells while reducing its toxicity in normal cells. A pronounced decrease in cell proliferation and enhanced apoptosis through the modulation of the mitochondrial membrane potential and key apoptotic regulators demonstrate the therapeutic potential of this combination. Our data suggest that the enhanced cytotoxicity observed is likely due to the activation of intrinsic apoptotic pathways, as evidenced by the increased expression of p53, caspase-3, and caspase-8, as well as mitochondrial depolarization in the treated tumor cells.

The involvement of TNF-α further suggests the activation of extrinsic apoptotic pathways, indicating a potentially dual mechanism of cell death. The selective action of AC-AFPK-IsCT1 in amplifying the effects of cisplatin on tumor cells, while mitigating the cisplatin-related adverse effect on normal cells, highlights its potential for improving the therapeutic index of conventional chemotherapies. This approach could lead to more effective treatment strategies for squamous cell carcinoma, potentially allowing for lower doses of cisplatin, thereby reducing the associated systemic toxicities. Further in vivo studies and clinical trials will be necessary to validate these findings and to explore the translational potential of this combination therapy.

## Figures and Tables

**Table 1:** IC50 values of cisplatin used as single agent or in combination with AC-AFPK-IsCT1 peptide in tumor cells (SCC-9 and SCC-25) and normal cells (FN1 and J774).

Treatment	Cell	IC50	Cell	IC50	Cell	IC50	Cell	IC50
Cisplatin 24 h	SCC-9	94.2	SCC-25	47.6	FN1	73.11	J-774	68.3
Cisplatin 48 h		5.2		15.2		3.06		8.7
Cis + AC-AFPK 50 µM 24 h		0.4		24.6		-		-
Cis + AC-AFPK 30 µM 24 h		19.9		31.7		-		-
Cis + AC-AFPK 10 µM 24 h		44.6		38.8		-		-
Cis + AC-AFPK 50 µM 48 h		0.8		4.7		36.21		48.57
Cis + AC-AFPK 30 µM 48 h		1.1		5.1		34		42.1
Cis + AC-AFPK 10 µM 48 h		1.3		6.7		31.8		37.5

(-) = did not show cytotoxicity. The fixed concentrations of the AC-AFPK-IsCT1 peptide were 50, 30, and 10 µM for SCC-9, FN1, and J-774 cells, and 40, 20, and 10 µM for SCC-25 cells, considering the higher peptide sensitivity of this cell line (Supplemental Figure S2).

**Table 2:** Treatments conducted on the tumoral SCC-9 and SCC-25 cells as well as the normal FN1 and J-774 cells.

	Treatment for SCC-9, FN1 and J-774 Cells
Group 1	Cisplatin
Group 2	Cisplatin (50 μM)/AC-AFPK-IsCT1 10 µM
Group 3	Cisplatin (50 μM)/AC-AFPK-IsCT1 30 µM
Group 4	Cisplatin (50 μM)/AC-AFPK-IsCT1 50 µM
	Treatment for SCC-25 cells
Group 5	Cisplatin
Group 6	Cisplatin (25 μM)/AC-AFPK-IsCT1 10 µM
Group 7	Cisplatin (25 μM)/AC-AFPK-IsCT1 20 µM
Group 8	Cisplatin (25 μM)/AC-AFPK-IsCT1 40 µM

Note: data from previous studies with the AC-AFPK-IsCT1 peptide have shown that concentration ranging from 10 to 50 μM did not cause harm to tumor or normal cells. In the current study, IC50 values for cisplatin and AC-AFPK-IsCT1, used as single agents or combined, were determined from the dose–response curves shown in Supplemental Figure S2.

## Data Availability

Data are contained within the articles.

## Supplementary Materials

The following supporting information can be downloaded at: https://www.mdpi.com/article/10.3390/molecules30122594/s1, Figure S1. LC/ESI-MS and analytical HPLC profiles of the purified AC-AFPK-IsCT1 peptide. (a) Electrospray ionization mass spectrometry (LC/ESI-MS) profile; (b) Analytical high-performance liquid chromatography (HPLC) chromatogram; (c) Table presenting the calculated and observed molecular masses, along with the purity levels determined from the chromatographic analysis of the AC-AFPK-IsCT1 peptide samples. Figure S2. Cytotoxicity assessment of treatments with the AC-AFPK-IsCT1 peptide and cisplatin, both individually and in combination. (a) Tumor and normal cells treated with AC-AFPK-IsCT1 peptide; (b) Tumor and normal cells treated with cisplatin for 24 h; (c) Tumor and normal cells treated with cisplatin for 48 h; (d) SCC-9 cells treated with the peptide–cisplatin combination; (e) SCC-25 cells treated with the peptide–cisplatin combination; (f) FN1 cells treated with the peptide–cisplatin combination; (g) J774 cells treated with the peptide–cisplatin combination. Line graphs show the cytotoxic effect expressed as mean ± SD from three independent experiments.
