# Supplementary material for: Evaluation of the Antitumor and Antiproliferative Potential of Synthetic Peptides Derived from IsCT1, Associated with Cisplatin, in Squamous Cell Carcinoma of the Oral Cavity
Source: Molecules. 2025 Jun 15;30(12):2594. doi: 10.3390/molecules30122594 (PMC12196148; doi:10.3390/molecules30122594)
Supplement: Supplementary file 1 [file molecules-30-02594-s001.zip › molecules-3644285-supplementary.pdf]

Supplementary Materials

Figure S1. LC/ESI-MS and analytical HPLC profiles of the purified AC-AFPK-IsCT1 peptide. (a) Electrospray ionization mass spectrometry (LC/ESI-MS) profile; **(b)** Analytical high-performance liquid chromatography (HPLC) chromatogram; **(c)** Table presenting the calculated and observed molecular masses, along with the purity levels determined from the chromatographic analysis of the AC-AFPK-IsCT1 peptide samples.

Print of window 80: MS Spectrum

(a)

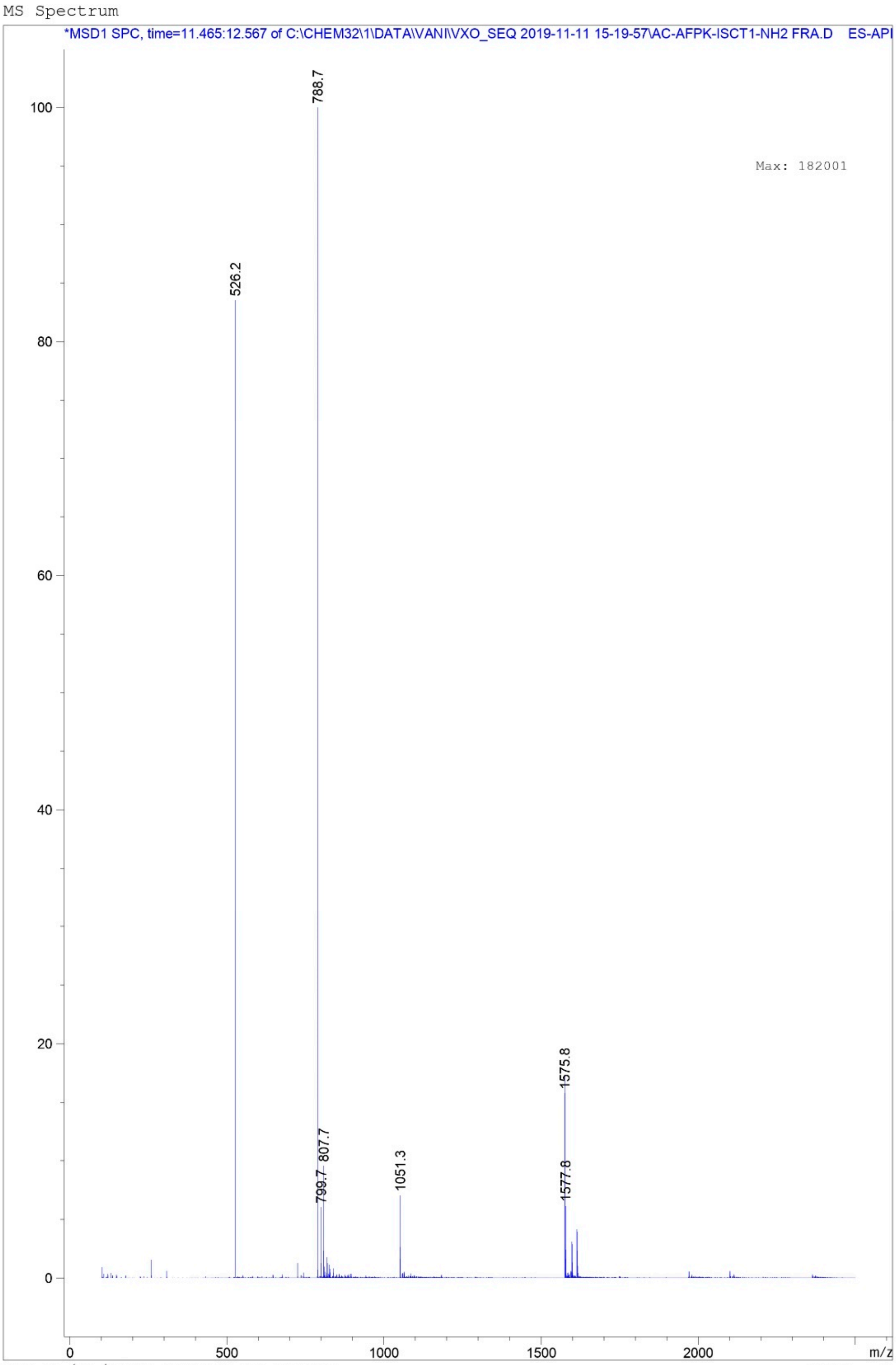

LCMS 6130B 11/11/2019 7:40:47 PM SYSTEM Page 1 of 1

(b)

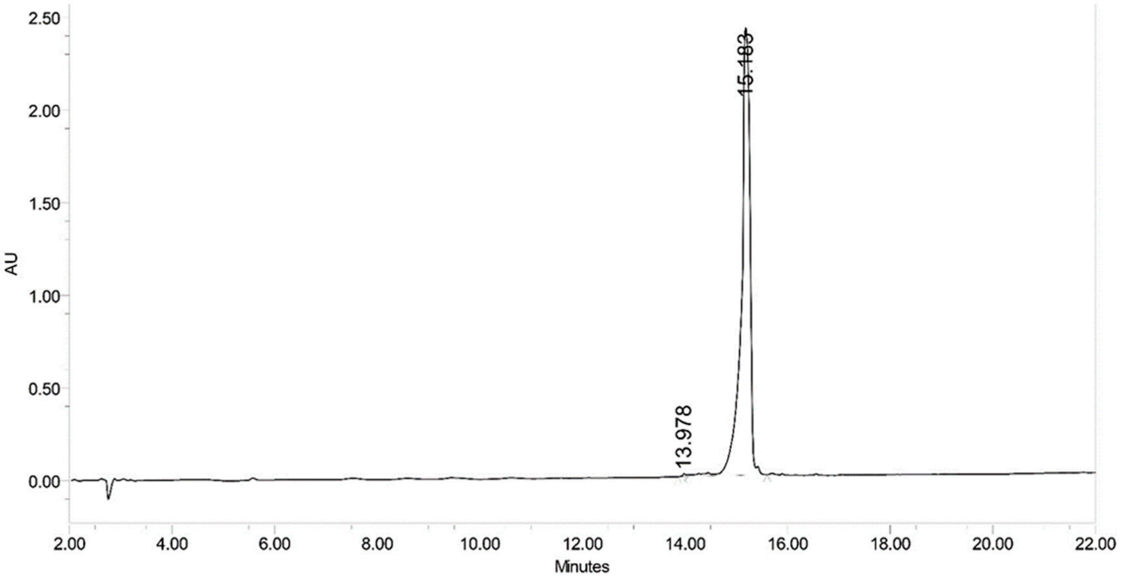

(c)

Peptide	Calculated	Observed mass
AC-AFPK-IsCT1	1575.9	1577.8
**Peptide**	**RT**	**Area (%)**
AC-AFPK-IsCT1	15.183	98%

Figure S2. Cytotoxicity assessment of treatments with the AC-AFPK-IsCT1 peptide and cisplatin, both individually and in combination. (**a**) Tumor and normal cells treated with AC-AFPK-IsCT1 peptide; **(b)** Tumor and normal cells treated with cisplatin for 24 h; **(c)** Tumor and normal cells treated with cisplatin for 48 h; (**d**) SCC-9 cells treated with the peptide–cisplatin combination; **(e)** SCC-25 cells treated with the peptide–cisplatin combination; **(f)** FN1 cells treated with the peptide–cisplatin combination; **(g)** J774 cells treated with the peptide–cisplatin combination. Line graphs show the cytotoxic effect expressed as mean ± SD from three independent experiments.

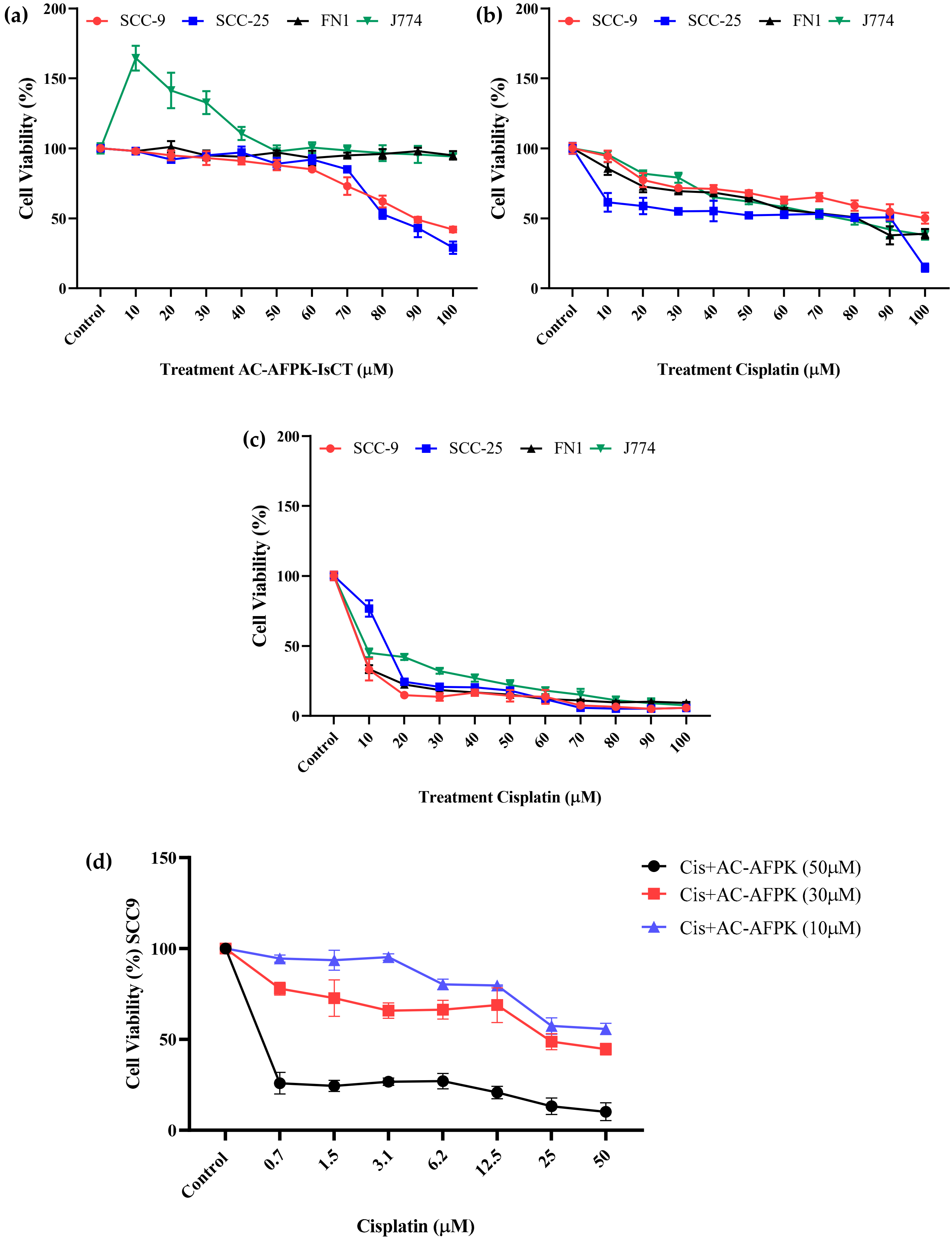
(a)
SCC-9
SCC-25
FN1
J774
Cell Viability (%)
200
150
100
50
0
Control
10
20
30
40
50
60
70
80
90
100
Treatment AC-AFPK-IsCT (μM)
(b)
SCC-9
SCC-25
FN1
J774
Cell Viability (%)
Treatment Cisplatin (μM)
(c)
SCC-9
SCC-25
FN1
J774
Cell Viability (%)
Treatment Cisplatin (μM)
(d)
Cis+AC-AFPK (50μM)
Cis+AC-AFPK (30μM)
Cis+AC-AFPK (10μM)
Cell Viability (%) SCC9
150
100
50
0
Control
0.7
1.5
3.1
6.2
12.5
25
50
Cisplatin (μM)

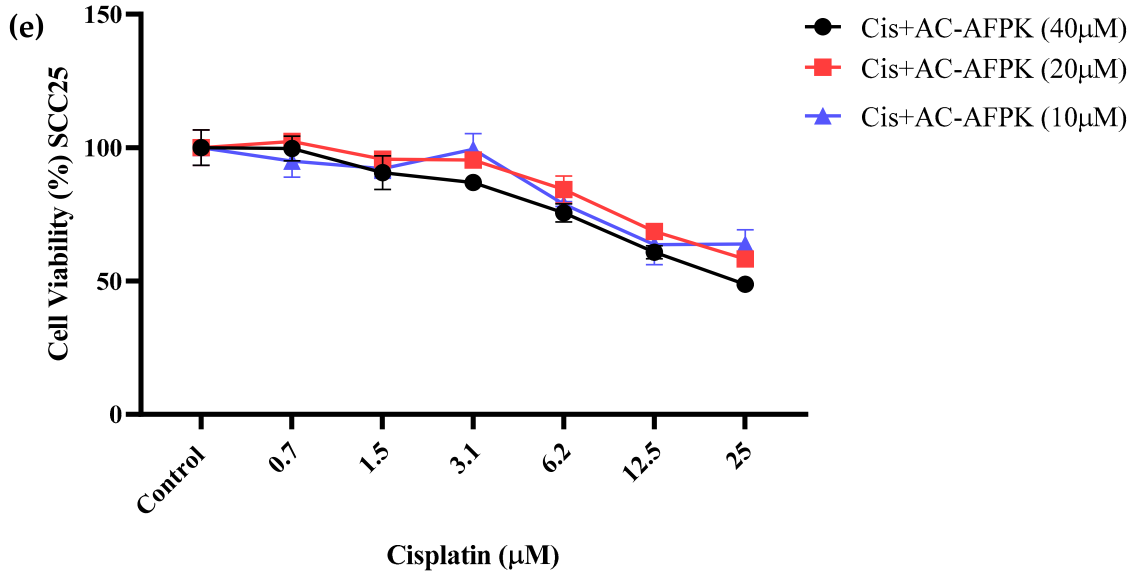
(e)
150
100
50
0
Cell Viability (%) SCC25
Control
0.7
1.5
3.1
6.2
12.5
25
Cisplatin (µM)
Cis+AC-AFPK (40µM)
Cis+AC-AFPK (20µM)
Cis+AC-AFPK (10µM)

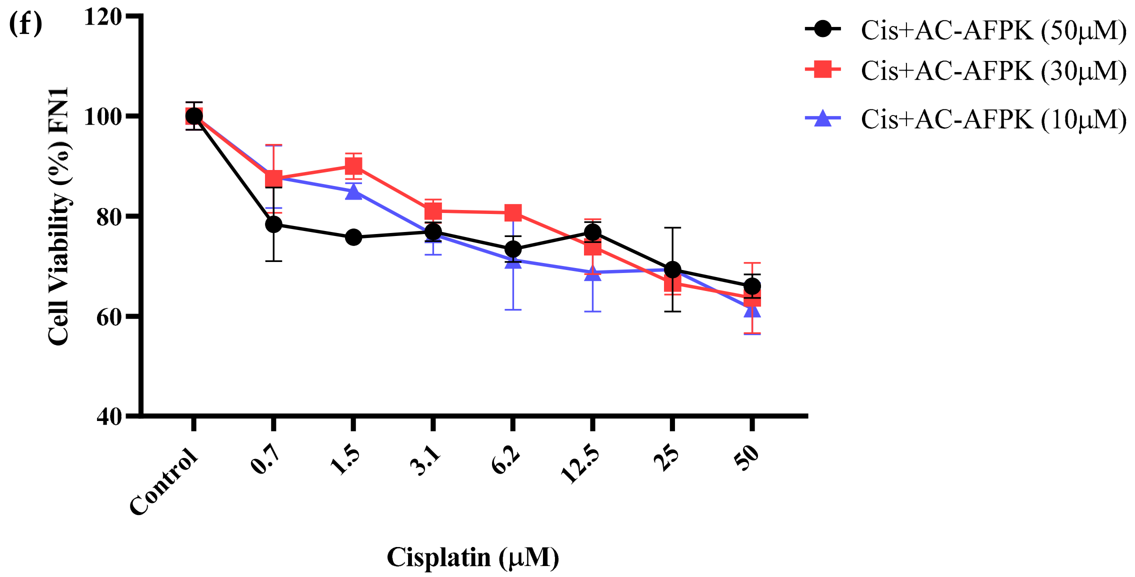
(f)
120
100
80
60
40
Cell Viability (%) FN1
Control
0.7
1.5
3.1
6.2
12.5
25
50
Cisplatin (µM)
Cis+AC-AFPK (50µM)
Cis+AC-AFPK (30µM)
Cis+AC-AFPK (10µM)

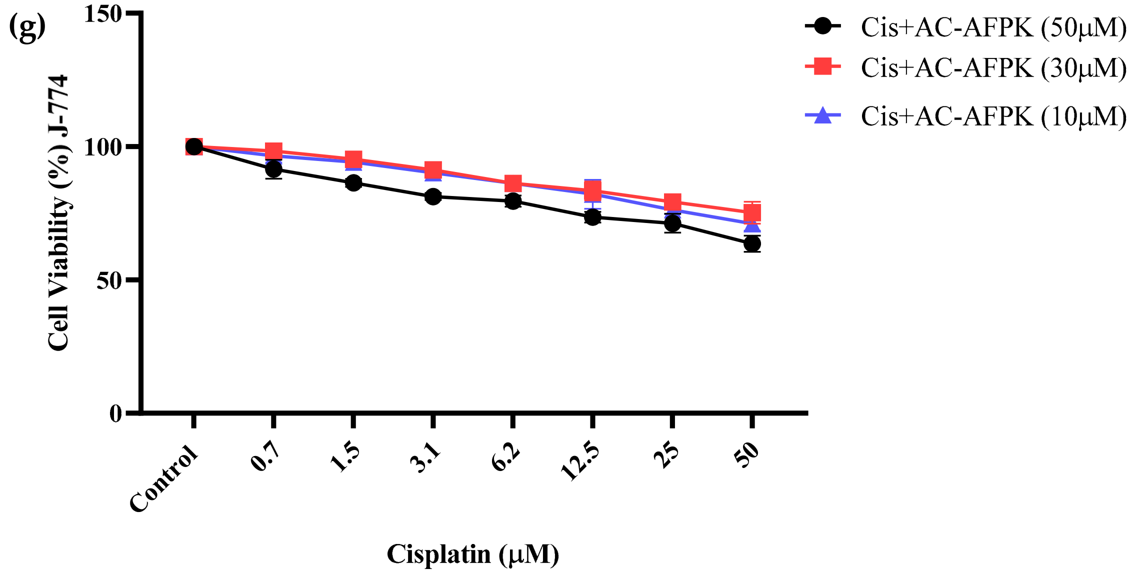
(g)
150
100
50
0
Cell Viability (%) J-774
Control
0.7
1.5
3.1
6.2
12.5
25
50
Cisplatin (µM)
Cis+AC-AFPK (50µM)
Cis+AC-AFPK (30µM)
Cis+AC-AFPK (10µM)
